# Preserved retinotopic brain connectivity in macular degeneration

**DOI:** 10.1111/opo.12279

**Published:** 2016-02-29

**Authors:** Koen V. Haak, Antony B. Morland, Gary S. Rubin, Frans W. Cornelissen

**Affiliations:** 1 Donders Institute for Brain, Cognition and Behaviour, Centre for Cognitive Neuroimaging, Radboud University, Nijmegen, The Netherlands; 2 York Neuroimaging Centre, Department of Psychology, University of York, York, UK; 3 Hull-York Medical School, York, UK; 4 Institute of Ophthalmology, University College London, London, UK; 5 Laboratory for Experimental Ophthalmology, University of Groningen, University Medical Center, Groningen, The Netherlands

**Keywords:** connective field modelling, cortical lesion projection zone, fMRI, macular degeneration, retinotopy

## Abstract

**Purpose:**

The eye disease macular degeneration (MD) is a leading cause of blindness worldwide. There is no cure for MD, but several promising treatments aimed at restoring vision at the level of the retina are currently under investigation. These treatments assume that the patient's brain can still process appropriately the retinal input once it is restored, but whether this assumption is correct has yet to be determined.

**Methods:**

We used functional magnetic resonance imaging (fMRI) and connective field modelling to determine whether the functional connectivity between the input-deprived portions of primary visual cortex (V1) and early extrastriate areas (V2/3) is still retinotopically organised. Specifically, in both patients with juvenile macular degeneration and age-matched controls with simulated retinal lesions, we assessed the extent to which the V1-referred connective fields of extrastriate voxels, as estimated on the basis of spontaneous fMRI signal fluctuations, adhered to retinotopic organisation.

**Results:**

We found that functional connectivity between the input-deprived portions of visual areas V1 and extrastriate cortex is still largely retinotopically organised in MD, although on average less so than in controls. Patients with stable fixation exhibited normal retinotopic connectivity, however, suggesting that for the patients with unstable fixation, eye-movements resulted in spurious, homogeneous signal modulations across the entire input-deprived cortex, which would have hampered our ability to assess their spatial structure of connectivity.

**Conclusions:**

Despite the prolonged loss of visual input due to MD, the cortico-cortical connections of input-deprived visual cortex remain largely intact. This suggests that the restoration of sight in macular degeneration can rely on a largely unchanged retinotopic representation in early visual cortex following loss of central retinal function.

## Introduction

Macular degeneration (MD) is an eye disease causing a progressive degeneration of the photoreceptor cells in the centre of the retina, and ultimately results in foveal vision loss. There is no cure for MD but several promising new treatments are currently under investigation.^1^ Many of those treatments are aimed at restoring the retinal signals, for example by using prosthetic implants, stem cell transplantation, or genetic therapy. Great progress has been made in developing these techniques, but it remains unclear whether the patient's brain is still capable of processing and interpreting the restored visual inputs after prolonged periods of visual deprivation.

Beside the technical difficulties of restoring the human retina, there are two reasons why visual recovery after prolonged visual deprivation might be problematic. First, it has been suggested that visual processing in input-deprived visual cortex undergoes large-scale reorganisation in some, but not all patients.^2^ That is, in the prolonged absence of visual stimulation, cortical neurons would shift their receptive fields toward the portions of the visual field that are still intact, thereby regaining visual sensitivity. Such changes would first need to be reversed before the restored inputs could be processed normally. However, more recent work indicates that large-scale remapping of visual cortex did not occur in a group of 16 MD patients.^3^ Second, the long-standing retinal pathology in MD has been associated with reductions in the white- and grey-matter density and volume along the input-deprived visual pathways.^4^ This suggests that long-term visual deprivation triggers visual cortical degeneration, which may in turn lead to irreversible damage to the visual cortical circuitry (see Prins *et al*.^7^ for a recent review). Thus, while it is largely reassuring that deprived primary visual cortex is generally not remapped,^8^ the reported anatomical changes in early visual cortex could still have adversely affected the functional cortico-cortical connections of the deprived cortex to areas downstream. In turn, this raises the question whether the visual brain would still be able to process appropriately retinal input—were this to be restored.

Previous work has already shown that the degeneration of input-deprived cortex is generally not sufficiently severe to fully abolish visual cortical activity. For example, transcorneal electrical stimulation still activates the input-deprived visual cortex in patients with retinal degeneration,^11^ suggesting that at least some portion of input-deprived visual cortex remains functional after prolonged visual deprivation. Furthermore, a recent case study has shown that the regions of visual cortex that have been deprived of sensory information by macular lesions can resume visually driven activity when retinal input is restored following anti-angiogenic treatment.^12^ These studies indicate that input-deprived visual cortex has at least some residual processing capabilities following visual deprivation in MD. However, cortical degeneration might still have disrupted the retinotopic configuration of visual cortex, which in turn could result in a distorted visual percept even if retinal function were restored. In this study, therefore, we revisited the data of Baseler *et al*.^3^ to evaluate explicitly the retinotopic configuration of input-deprived visual cortex in MD.

Cortical reorganisation in the form of remapping is characterised by cells that retained or regained visual sensitivity, whereas cortical degeneration is characterised by the death of cells that did not. As such, the presence or absence of cortical remapping can be tested using visual stimulation, but the impaired integrity of the visual cortical circuitry due to cortical degeneration cannot. We therefore employed connective field modelling,^13^ a new functional MRI data-analysis tool that extends the procedure of estimating a voxel's population receptive field (pRF)^14^ towards estimating a voxel's connective field. Just as a voxel's pRF predicts its activity as a function of stimulus position, its connective field predicts the activity as a function of activity in another part of the brain. That is, whereas finding the pRF of a voxel involves estimating the location and width parameters of a stimulus-referred Gaussian receptive field model defined in visual space, the connective field of a voxel in one brain area (e.g., V2) is found by estimating the location and width parameters of a neural-referred Gaussian receptive field model that follows the cortical surface of another brain area (e.g., V1). Conceptually, this means that the activity elsewhere in the brain acts as the stimulus for that voxel. Unlike the stimulus-referred pRF, therefore, the neural-referred connective field can also account for brain activity that occurs in the absence of visual stimulation.^13^ Using connective field modelling, therefore, we asked whether or not the extrastriate cortex connective fields within primary visual cortex were disrupted in patients with MD compared with controls in whom we simulated scotomas.

## Methods

As we revisited the data of Baseler *et al*.^3^ several aspects of the Methods (i.e., *Magnetic Resonance Imaging*, *Retinotopic Mapping*, and *Data Preprocessing*) are the same as those described in that publication and have therefore been reproduced here with only minor modifications.

### Subjects

Eight individuals with MD (Table 1) were recruited at the Moorfields Eye Hospital, London. All of them had established bilateral lesions for at least 1 year, with a central scotoma of less than 10° radius spanning the fovea and a stable preferred retinal locus. This was our entrance criterion for a larger study^3^ on both age-related and juvenile MD. The present analysis was limited to the patients with juvenile MD to allow for an age-matched comparison between patients and controls with simulated lesions; although the dataset presented in Baseler *et al*.^3^ consisted of elderly and younger controls, only the younger controls were tested with stimuli that simulated lesions. The majority of the juvenile MD patients reported on here had lesions that had been established for far longer than 1 year. The patients had Stargardt's disease that usually leads to loss of macular vision in early adulthood. Visual field sensitivity and fixation stability for all MD patients were evaluated directly on the retina using an MP1 microperimeter (NIDEK; www.nidek-intl.com). The preferred retinal locus (PRL) coordinates and fixation stability (bi-variate contour ellipse area, BCEA) were determined using methods outlined elsewhere.^18^ Twelve age-matched control participants (ages 18–41) were recruited at the York Neuroimaging Centre for an experiment using simulated retinal lesions. All control participants had normal or corrected-to-normal vision. Experimental procedures were approved by the London Multicentre Research Ethics Committee, Royal Holloway University of London Ethics Committee and the York Neuroimaging Centre Science and Ethics Committee, and adhered to the tenets of the Declaration of Helsinki.

**Table 1:** Summary of patients in the study. Acuity (logMAR) measures the minimum angle of resolution. The bivariate contour ellipse area (BCEA) is a measure of fixation stability using microperimetry

Patient	Sex	Age (years)	Eye tested	Lesion diameter (°)	Acuity (logMAR)	BCEA (°)
1	M	19.8	Left	5	0.74	11.71
2	F	19.7	Left	3	1.02	2.24
3	M	49.5	Right	6	0.56	14.47
4	F	41.2	Right	10	0.90	1.69
5	F	34.7	Right	8	1.08	13.54
6	F	39.4	Right	9	0.98	18.11
7	F	24.3	Left	3.5	0.66	9.33
8	M	35.8	Left	17	1.12	13.26

### Magnetic resonance imaging

Functional MRI and structural MRI data were acquired using 8-channel, phase-array head coils on either a Siemens Trio 3 Tesla at the Combined Universities Brain Imaging Center (CUBIC, Royal Holloway University of London), or on a GE 3-Tesla Signa HD Excite scanner at the York Neuroimaging Centre (YNiC, University of York). For structural data, multi-average, whole-head T1-weighted anatomical volumes were acquired for each participant (1.0 × 1.0 × 1.13 mm^3^). Sequences used were 3D-MDEFT on the Siemens Trio (www.healthcare.siemens.com) or 3D-FSPGR on the GE Signa (www.gehealthcare.com); imaging parameters in both sequences provide good grey-white contrast allowing the segmentation of anatomical data into grey and white matter, and subsequent visualization in volume and inflated cortical views. For functional data, gradient recalled echo pulse sequences were used to measure T2* blood-oxygen level dependent (BOLD) data (repetition time = 3 000 ms, echo time = 30 ms, field of view = 28.8 cm, 128 × 128 matrix, 25 contiguous slices with 3-mm slice thickness). Images were read out using an EPI sequence. Magnetisation was allowed to reach a steady state by discarding the first five volumes.

### Retinotopic mapping

Computer-generated visual stimuli were presented using a LCD projector (Sanyo PLC-XP40L at CUBIC; www.sanyo-projectors.co.uk, Dukane ImagePro 8942 at YNiC; www.dukane.com). Stimuli were rear projected onto an acrylic screen situated in the bore of the MRI scanner, behind the participant's head. Participants viewed the stimuli monocularly (i.e., with one of their eyes covered with a patch) via a mirror mounted on the head coil. Stimuli were generated with MATLAB (www.mathworks.com) and controlled by MatVis (Neurometrics Institute, Berkeley, CA). All stimuli were unmasked portions of a 100% contrast radial checkerboard with 8 rings and 24 radial segments on a mean grey background. Contrast reversal rate was 6 Hz. Stimulus size was 30 by 30° of visual angle. The stimulus comprised three rings of the checkerboard that increased in angular extent. As it moved out from the centre of the visual field, a new ring at the centre replaced an existing ring as it approached the edge of the visual field. The stimulus had a period of 36 s and was repeated for seven full cycles. A red fixation cross was placed at each patient's stable PRL. Four data sets were typically collected for each MD patient. The 12 control participants were shown a masked version to simulate a central lesion. The mask consisted of a centrally placed static disk (7.5° radius) at mean luminance grey such that the central portion of the visual field was constant throughout the scan. A red fixation cross was placed in the centre of the stimulus and at least two scans were acquired per control subject.

### Data preprocessing

Data were analysed using the mrVISTA toolbox (http://white.stanford.edu/software/). For the anatomical data, the occipital cortices of the acquired anatomical volumes were manually segmented into white and grey volumes. The grey-matter surface of each subject was constructed and subsequently rendered in three dimensions. For functional data, images were corrected for spatial inhomogeneity and motion corrected. Baseline drifts were removed using a discrete cosine transform high-pass filter. Percent signal change was computed for each voxel by subtracting and dividing by its mean amplitude value over time. The fMRI data were manually aligned to the high-resolution anatomical volume.

### V1 definition

**Figure 1:**
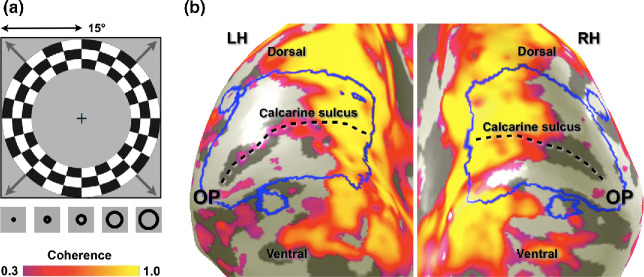
ROI definitions. (a) The expanding ring stimulus that was used to identify the input-deprived regions of visual cortex. The maximum stimulus radius was 15° of visual angle. The thumbnail images at the bottom of the panel show how the stimulus changed over time (time runs from left to right). The same stimulus prescription was used for the control participants but here the central 7.5° of the visual field was masked so that it always showed the same mean luminance grey as the background. (b) Portions of visual cortex that demonstrated stimulus-synchronised activity with a coherence value larger than 0.3. Here we defined the input-deprived portions of visual cortex to comprise all voxels that did not show such stimulus-synchronised activity. In both patients and controls we defined visual area V1 on the basis of anatomical criteria – the larger blue contours surrounding the calcarine sulcus. Furthermore, four circular extrastriate ROIs were defined inside input-deprived visual cortex: one dorsally and one ventrally to V1 in the left hemisphere of the brain (normally responding to the lower and upper right visual quarter-fields, respectively), and one dorsally and one ventrally to V1 in the right hemisphere of the brain (normally responding to the lower and upper left visual quarter-fields, respectively). V1 connective fields were estimated for all voxels (with a stimulus-response coherence less than 0.3) residing inside these four extrastriate ROIs.

Because it is impossible to visually stimulate the regions of cortex representing the centre of the visual field in MD, within these regions the boundaries of visual area V1 cannot be functionally identified using standard retinotopic mapping. However, a limited set of anatomical landmarks and cortical folding patterns can be used to define the V1 boundaries for an individual subject with the same precision as 10–25 minutes of retinotopic mapping.^19^ Therefore, the V1 boundaries were drawn manually on a three-dimensional surface reconstruction of the boundary between the segmented grey- and white-matter volumes on the basis of the following anatomical criteria: the V1 boundaries followed the gyral convexities surrounding the calcarine sulcus from the parietal-occipital fissure to the occipital pole via the cuneate gyrus and back via the lingual gyrus (*Figure* 1). The V1 ROIs consisted of all voxels enclosed by the V1 boundaries that belonged to the contiguous grey matter directly adjacent to the white-matter volume. The same procedure was applied in both the MD patients and controls for the left and right cerebral hemispheres, thus creating two V1 ROIs that—for computational purposes—were subsequently combined into a single V1 ROI per subject.

### Extrastriate ROIs

We first assessed the strength of the stimulus-synchronized activity at each voxel using its coherence value, which is defined as the Fourier amplitude of the BOLD signal at the stimulus fundamental frequency (f_0_ = 7 cycles per scan) divided by the square root of the time series power.^20^ To this end, functional data were averaged across scans for repeated scans within a session for each individual. The input-deprived portions of extrastriate cortex were then defined by all voxels outside V1 with a coherence value under 0.3 (*Figure* 1). We next defined four ROIs, one for each of the quarter-field maps of visual area V2/V3, by gathering all contiguous grey matter voxels within circular patches, 4 mm radius, each centred on a manually selected point outside the V1 ROIs but comfortably inside the cortical lesion projection zone. We refer to these ROIs as ‘V2/V3’ because there is some uncertainty about whether the ROIs fall entirely within V2 or partially overlap with V3. Voxels with coherence values greater than 0.3 were always explicitly excluded from the extrastriate ROIs. Just as the same procedure was taken to define V1 in both MD patients and controls, the same procedure was taken to define the extrastriate ROIs in both subject groups.

### Connective field modelling

Connective fields were estimated^13^ from the un-averaged fMRI time-series data. This allows estimating the connective field properties based on spontaneous brain activity that would otherwise be averaged out. The un-averaged fMRI time-series were filtered using a tenth order low-pass Butterworth filter with a cut-off frequency of 0.1 Hz to reduce the influence of non-neuronal physiological noise.^21^ The fMRI response of each voxel in each of the four extrastriate ROIs was predicted using a two-dimensional circular symmetric Gaussian connective field model, folded to follow the cortical surface of the V1-ROI (all V1 grey-matter voxels directly adjacent to the white-matter). We determined the mapping of the functional connections from V1 (comprising both hemispheres) to each of the dorsal and ventral extrastriate ROIs in both hemispheres. The free parameters of these connective field models are the connective field position and the Gaussian spread across the V1 surface. The optimal model parameters were found by minimising the residual sum of squares between the model's time-series prediction and the observed time-series. To achieve this, a wide range of time-series predictions were generated by varying the connective field position across all existing voxel positions on the V1 surface (note that the V1 surface comprised voxels from both cerebral hemispheres) and 50 Gaussian spread values up to 25 mm (0.5 mm steps) across the cortical surface of V1. As in previous work,^13^ best models were retained if the explained variance in the observed fMRI time-series exceeded 15%.

### Evaluating retinotopic configuration

We concentrated our analyses on detecting polar-angle preserving connectivity patterns, because the patients we studied had retinal lesions that varied in size, which precludes group estimates of eccentricity preserving connectivity patterns; polar-angle is represented at all eccentricities and can therefore be assessed at the group level even when the size of the retinal lesion varies. In addition, because the data from Baseler *et al*.^3^ were gathered while subjects viewed expanding ring-stimuli outside the (simulated) scotomas, the peripheral ring-stimuli may have modulated the fMRI activity in input-deprived visual cortex.^3^ However, if so, they would have done so equally across all polar-angles, thereby providing no experimentally induced information on the basis of which polar-angle preserving connectivity might be detected. Note also that the peripheral ring stimuli impede estimating the representation of polar angle outside the LPZ, thereby precluding within-subject comparisons between the deprived and non-deprived portions of visual cortex. As such, they prevent connective field model solutions with locations outside the V1 LPZ, because the spontaneous signal fluctuations from within the extrastriate LPZ ROIs do not fit well with stimulus induced waves of activity.

If functional connections between V1 and extrastriate cortex are retinotopically configured, then the connective fields of voxels in the left and right dorsal extrastriate ROIs should be positioned at the upper bank of the calcarine sulcus of the left and right hemisphere, respectively. Likewise, the connective fields of voxels in the left and right ventral extrastriate ROIs should be located at the lower bank of the calcarine sulcus of the left and right hemisphere, respectively. In other words, if functional connections between V1 and extrastriate cortex are retinotopically configured, then the expected connective field positions for a voxel in a certain extrastriate ROI are those that are closest—in terms of the distance across the folded cortical surface—to that particular ROI. Therefore, for each subject, we counted how many voxels in each extrastriate ROI had a connective field positioned closest to itself or to any of the three other extrastriate ROIs. We organised these scores into a 4 × 4 connectivity matrix, with columns indicating the sampled extrastriate ROI, and rows representing the connective field locations in V1. To assess the degree of retinotopic configuration in each subject, we used Cohen's kappa coefficient^23^ to quantify the agreement between the estimated and the expected connective field positions. Cohen's kappa was computed using the following formula: (*p*_0_ − *p*_c_)/(1 − *p*_c_), where *p*_0_ is the percentage agreement between the expected and estimated connective field locations (i.e., the sum of the diagonal cells in the connectivity matrix divided by the sum across all cells), and *p*_c_ the percentage chance agreement to account for the agreement that would have occurred by chance. The percentage chance agreement, *p*_c_, is given by (**1**^T^ ∙ X) ∙ (X ∙ **1**), where X contains the scores in the connectivity matrix divided by the total number of connective field centres, and **1** is a column vector of ones. Cohen's kappa ranges between 0 (no agreement) and 1 (total agreement) and was computed for each subject separately (and group averaged).

## Results

**Figure 2:**
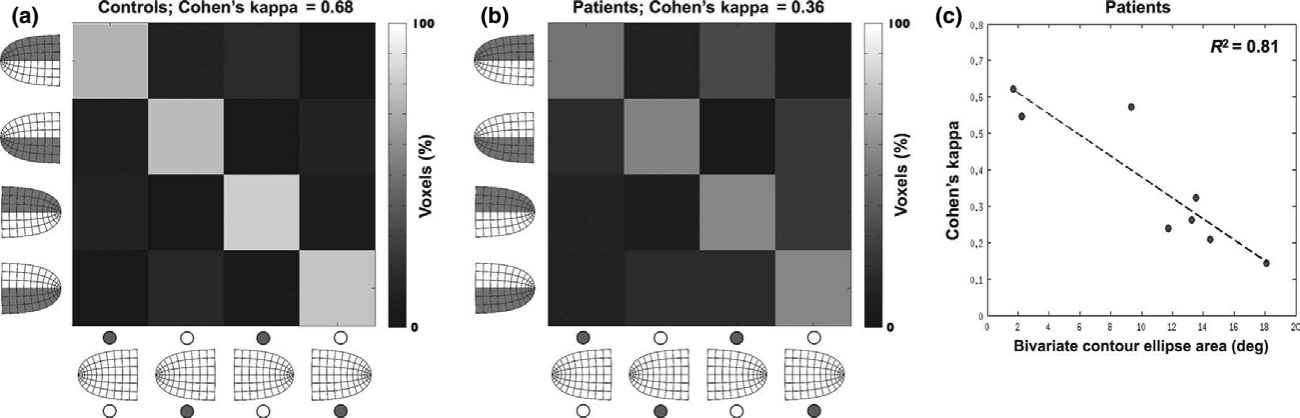
Results. (a) Average connectivity matrix for the healthy controls with simulated lesions. Columns indicate the sampled region of extrastriate cortex. Rows indicate connective field locations in V1. Diagonal bins indicate agreement between the expected and the estimated connective field location, which is in turn indicative of topographic connectivity between visual areas V1 and extrastriate cortex. It can be seen that topographic connectivity can be traced despite the absence of visual input in the sampled areas of visual cortex (high counts for the diagonal bins). It can also be seen that if confusions occurred, they most often were between the expected portion of V1 and its contra-lateral counterpart, indicating that confusions were based on retinotopic distance rather than on cortical distance. Percentages were computed per row (rows add up to 100%). (b) Average connectivity matrix for the MD patients. Note that despite the decreased kappa value, there still is substantial agreement between the expected and estimated connective field locations. (c) The kappa values for the patients are strongly dependent on the bi-variate contour ellipse area (BCEA), which measures fixation instability. Note that the dotted regression line intersects the *y*-axis at about the same kappa value as the average kappa value for the controls.

We first evaluated whether connective field modelling was capable of tracing retinotopic connectivity within the input-deprived portions of visual cortex in healthy controls with simulated lesions. If input-deprived V1 exhibits polar-angle preserving connectivity, then the connective field centres of voxels within the dorsal-right extrastriate ROI should be located in dorsal right V1, whereas the connective field centres of voxels within the ventral-right extrastriate ROI should be located in ventral right V1, and a similar pattern should be observed in the left hemisphere. *Figure* 2*a* shows that this expected pattern is indeed observed, because the majority of voxels fall in the diagonal bins of the connectivity matrix. The connective field estimates underlying this result were very reliable, explaining on average 60% of the variance in the time-series. The agreement between the expected and estimated connective field locations shown in *Figure* 2*a* (diagonal cells in the connectivity matrix) is further demonstrated by a highly significant mean (across subjects) Cohen's kappa value (M = 0.68, S.D. = 0.2, *t*_11_ = 12.0, *P* < 0.0001).

*Figure* 2*a* also shows that the dorsal and ventral extrastriate ROIs appear to exhibit significant connectivity with their dorsal and ventral V1 counterparts, but in the opposite hemisphere (as seen in the off-diagonal bins). As before, the connective fields underlying these connections were very reliable, explaining 48% of the variance in the time-series on average. It is likely that this connectivity pattern is underpinned by the inputs that extrastriate neurons (with receptive fields near the vertical meridian) receive from the opposite hemisphere^24^ (see Haak *et al*.^13^ for a discussion of the effect on connective field estimates). Indeed, extrastriate voxels with connective fields in the opposing hemisphere tended to be closer to the V1 border—the cortical representation of the vertical meridian—than the voxels that exhibited the expected pattern of connectivity (*t*_856_ = 1.60 *P* = 0.05; distance measured along the cortical surface). Therefore, it appears that the proximity of the ROIs in visual space drives the connectivity. However, it is necessary to discount other proximity effects based on the volumetric separations of the ROIs. The volumetric distances between the four V1 sub-regions were much greater between- vs within-hemispheres (*t*_22_ = 8.76, *P* < 0.0001), and could therefore not explain the inter-hemispheric connectivity patterns of the connectivity matrix (*R*^*2*^ = 0.04, *P* = 0.52; comparison of the pairwise volumetric distances between the four V1 sub-regions against the voxel counts in the off-diagonal cells of the connectivity matrix). Thus, it is unlikely that the inter-hemispheric connectivity was determined by potentially confounding factors such as BOLD signal smearing. Given that the BOLD spread for this type of experiment^20^ is considerably smaller than the volumetric distance of ~11 mm between the correctly classified extrastriate voxels and the centres of their connective fields in V1, BOLD smearing can also be ruled out as an explanation for the correct, on-diagonal results. Finally, the results cannot be explained by head motion or differences in BOLD amplitude, as we found no relationship between Cohen's kappa and head motion (*R*^*2*^ = 0.07, *P* = 0.38), or BOLD amplitude (*R*^*2*^ = 0.15, *P* = 0.2). Thus, in agreement with previous work,^15^ this confirms that the connective field modelling approach is capable of tracing retinotopic connectivity, both within and between hemispheres, in the absence of stimulus driven activity.

We next assessed whether the MD patients exhibited a similar agreement between the expected and estimated connective field locations as the controls with simulated lesions. Again, the connective field estimates were highly reliable, explaining on average 53% and 44% of the time-series variance for the on- and off-diagonal voxels, respectively. As illustrated in *Figure* 2*b*, we also found a highly significant mean kappa value (M = 0.36, S.D. = 0.19, *t*_7_ = 5.53, *P* < 0.0001) for the MD patients, indicating that there is at least some residual retinotopic configuration after prolonged visual deprivation. As for the control participants, the differences between expected and estimated connective field locations were mainly due to confusions between hemispheres, while the volumetric distances between the four V1 sub-regions were much greater between- vs within-hemispheres (*t*_14_ = 5.73, *P* < 0.0001), and could therefore not explain the inter-hemispheric connectivity (*R*^2^ = 0.17, *P* = 0.18). Also, for the correctly classified extrastriate voxels, the average volumetric distance to the centres of their connective fields was ~14 mm—much greater than the BOLD spread. Still, the kappa values for the MD patients were significantly different when compared directly with the kappa values for the controls (*t*_18_ = 3.61, *P* = 0.002). A two-way anova (factors: group and ROI) further revealed that this difference could not be due to a bias in the cortical distance between V1 and the extrastriate ROIs, since there was no significant main effect of group (*F*_1,72_ = 0.69, *P* = 0.41), no significant main effect of ROI (*F*_3,72_ = 0.72, *P* = 0.54), and no significant interaction between them (*F*_3,72_ = 1.56, *P* = 0.21). The difference between patients and controls, however, could be explained on the basis of fixation instability. *Figure* 2*c* shows that there is a significant relationship between kappa and the patients’ fixation stability; there is a highly significant slope of the regression line between kappa and the bi-variate contour ellipse area (BCEA; *R*^2^ = 0.81, *P* < 0.0001). Importantly, also, the *y*-intercept of this regression line, which amounts to 0.67, adheres closely to the mean kappa value for the control subjects of 0.68. We did not find a significant relationship between kappa and the patients’ lesion diameter (*R*^*2*^ = 0.09, *P* = 0.47), head motion (*R*^*2*^ = 0.03, *P* = 0.68), or BOLD amplitude (*R*^*2*^ = 0.12, *P* = 0.40), nor a significant difference between patients and controls for the connective field spread (*t*_18_ = 0.55, *P* = 0.59).

## Discussion

Using connective field modelling, we found retinotopically organised patterns of functional connectivity in the cortical lesion projection zone of patients with bilateral retinal lesions due to MD. This is also revealed in control subjects when a retinal scotoma is simulated. The results of both groups show that it is feasible to assess the integrity of visual cortical connectivity from BOLD signals that arise spontaneously, rather than from those driven by stimuli. This agrees with previous work that derived retinotopic and connective field maps in the complete absence of visual stimulation.^15^

On average, MD patients with a genuine loss of vision had a less clear pattern of retinotopic connectivity than control subjects in whom loss of vision was simulated. It is possible that this difference arises because the changes in visual cortical structure seen in patients with MD^4^ may in turn affect the precision of the neural representation and connectivity. However, previous work has shown that such changes in cortical structure are correlated with retinal lesion size.^5^ If a link existed between decreased connectivity and cortical degeneration, connectivity and lesion size should be similarly related. That we did not find such a relationship argues against a strong influence of cortical degeneration on the visual connectivity patterns in MD.

MD patients with good fixation stability had patterns of connectivity that were no different from those of controls. This suggests that eye-movements caused the observed group-difference (though it should be noted that in the absence of fixation stability measurements in the control group, we could not test this explicitly). If large eye-movements are made, a region of the peripheral visual field that is initially visible to the patient will fall within the scotoma following the eye-movement. Potentially, this could give rise to a prediction mismatch,^25^ which in turn could lead to a BOLD signal across the entire cortex that represents the scotoma.^26^ Such a signal with a largely uniform spatial structure would hamper our ability to assess the spatial structure of connectivity. Importantly, previous work has not shown a link between fixation stability and grey matter reductions in early visual cortex,^5^ so further experiments would be required to tease apart the effects of cortical degeneration and eye-movements on measures of cortical connectivity.

One might also entertain the hypothesis that the correlation between the lowered kappa values for patients and fixation stability was caused by cortical reorganisation. Following the onset of retinal lesions, fixation stability improves over time^5^ and some form of cortical reorganisation could quite plausibly underpin this. However, if reorganisation of the topographic relationships between V1 and extrastriate cortex underpinned the improvement of fixation stability, we would expect to find reduced spatial structure in the connectivity patterns. We find the opposite: stable fixation equates to normal connectivity patterns. Thus, our data do not support this hypothesis.

In principle, we could have based our analyses on simple point-to-point correlations rather than connective field modelling. We elected to employ connective field modelling because we expected it to be more sensitive than a simple point-to-point correlation analysis. This is because connective field modelling allows for the combined activity of an extended area of cortex to explain the activity in another visual area. Under normal retinotopic mapping stimulation, this extended area of V1 better explains the activity patterns of voxels within higher visual areas than single V1 voxels (or the connective field estimates under those conditions would have encompassed just a single voxel). In addition, connective field modelling effectively reduces to a simple point-to-point correlation analysis in cases where there is no indication that more than just one V1 voxel is required to adequately explain the time-series variance of voxels in higher order visual areas.

It also would have been possible to base our analyses on connectivity measurements obtained with pure resting-state fMRI. That is, the present fMRI signals from the LPZ should be effectively the same as the signals that can be obtained with eyes-closed resting-state fMRI, though the latter would not be potentially influenced by the peripheral stimulus ring stimuli that were present when the present dataset was recorded. In order to minimise these influences, we took care to define the regions of interest comfortably within the margins of the lesion projection zone. That this approach was indeed effective follows from the fact that we were able to trace retinotopic connectivity in non-stimulated cortex at all. That is, we would not have been able to distinguish connectivity patterns within the polar-angle domain if the ring-stimuli had had a large influence on the fMRI signals in the regions of interest. This is because the ring-stimuli produce correlations across iso-eccentricity bands, which hampers the ability to distinguish connectivity in the polar-angle domain. That said, in our decision to base our analyses on the presented dataset we did not anticipate the possibility that the presence of peripheral stimulation could cause the patient group to exhibit slightly decreased patterns of retinotopic connectivity due to prediction errors generated by instable fixation. This effect would have likely been absent had we based our analysis on pure resting-state scans. However, the fact remains that we could still detect retinotopic connectivity in the patients, and that patients with stable fixation exhibited patterns of connectivity that were indistinguishable from those observed in controls. Thus, our conclusion would have been the same: MD patients still exhibit intact retinotopic connectivity, even after years of visual deprivation.

The main limitation of our study is that we only evaluated the relatively coarse retinotopic connectivity patterns that normally exist between V1 and four extrastriate ROIs. We were constrained to do so, because the same extrastriate regions had to be identified in each participant such that they were of equal size and fell within sensory deprived patches of cortex. Given that the input-deprived area of visual cortex varies across individuals, we were required to select a relatively posterior portion of extrastriate cortex on anatomical grounds in each participant, to allow those patients with relatively small input-deprived areas to be included. At these locations, it would be very challenging to look at anything other than relatively coarse retinotopic connectivity patterns. Future work focusing on a more homogeneous group of MD patients with relatively large retinal lesions and normal fixation stability may be able to confirm the sustained presence of finer grained retinotopic connectivity patterns in patients with MD.

We conclude that the retinotopic representation in early visual areas of the human brain remains largely present even after a prolonged loss of visual input. This indicates that if retinal function were to be restored, the brain is probably still appropriately configured to process the restored input. However, this is not to say that nothing has changed. The reductions in grey and white matter seen in MD patients^4^ could still mean that restoring retinal signals may not result in an immediate and complete restoration of normal macular vision.

## Disclosure

The authors report no conflicts of interest and have no proprietary interest in any of the materials mentioned in this article.
